# Resveratrol-Induced Effects on Body Fat Differ Depending on Feeding Conditions

**DOI:** 10.3390/molecules22122091

**Published:** 2017-11-29

**Authors:** Iñaki Milton-Laskibar, Saioa Gómez-Zorita, Leixuri Aguirre, Alfredo Fernández-Quintela, Marcela González, María P. Portillo

**Affiliations:** 1 Nutrition and Obesity Group, Department of Nutrition and Food Science, Faculty of Pharmacy and Lucio Lascaray Research Center, University of the Basque Country (UPV/EHU), 01006 Vitoria, Spain; inaki.milton@ehu.eus (I.M.-L.); saioa.gomez@ehu.eus (S.G.-Z.); leixuri.aguirre@ehu.eus (L.A.); alfredo.fernandez@ehu.eus (A.F.-Q.); 2 CIBEROBN Physiopathology of Obesity and Nutrition, Institute of Health Carlos III (ISCIII), 28029 Madrid, Spain; 3 Nutrition and Food Science Department, Faculty of Biochemistry and Biological Sciences, National University of the Littoral and National Council of Scientific and Technological Research (CONICET), 3000 Santa Fe, Argentina; maidagon@fbcb.unl.edu.ar

**Keywords:** resveratrol, normal feeding, overfeeding, energy restriction, rodents, human

## Abstract

Science constantly seeks to identify new molecules that could be used as dietary functional ingredients in the fight against obesity and its co-morbidities. Among them, polyphenols represent a group of molecules of increasing interest. One of the most widely studied polyphenols is resveratrol (*trans*-3,4′,5-trihydroxystilbene), which has been proposed as an “energy restriction mimetic” because it can exert energy restriction-like effects. The aim of this review is to analyze the effects of resveratrol on obesity under different feeding conditions, such as overfeeding, normal feeding, and energy restriction, in animals and humans. The vast majority of the studies reported have addressed the administration of resveratrol to animals alongside an obesogenic diet. Under these experimental conditions usually a decreased body weight amount was found. To date, studies that focus on the effects of resveratrol under normal feeding or energy restriction conditions in animals and humans are scarcer. In these studies no changes in body fat were reported. After analyzing the results obtained under overfeeding, normal feeding, and energy restriction conditions, it can be stated that resveratrol is useful in reducing body fat accumulation, and thus preventing obesity. Nevertheless, for ethical reasons, these results have been obtained in animals. By contrast, there are no evidences showing the usefulness of this phenolic compound in reducing previously accumulated body fat. Consequently, as of yet, there is not scientific support for proposing resveratrol as a new anti-obesity treatment tool.

## 1. Introduction

Obesity is a multifactorial, chronic disease that is characterized by excessive body fat accumulation. It is considered to be a risk factor in the development of various diseases, which include hypertension, type 2 diabetes, coronary heart disease, and respiratory complications, among others [1,2]. Moreover, it has become a major health problem in the Western societies, where it has reached epidemic proportions [3]. In the latest data published by the WHO, more than 1.9 billion adults, aged 18 and older, were overweight. Of these, over 650 million were obese. It is important to take into account that obesity is not only an adult problem, as children are also increasingly affected. As far as children are concerned, over 340 million children and adolescents aged 5–19 were overweight or obese in 2016 [3].

Obesity is considered as a multifactorial disease, which is influenced by lifestyle, cultural, environmental, genetic, physiological, and metabolic factors. Notably, non-balanced dietary patterns and reduced physical activity are two main factors leading to the increased prevalence of obesity. Energy restriction is a non-pharmacological intervention, which is commonly used to treat obesity and its co-morbidities [4,5,6]. Basically, this treatment consists of a reduction of 20–40% of the total daily energy intake, without inducing malnutrition [7,8]. Compliance with this dietary pattern is an important issue in the chances of the long-term success of this approach. However, due to social, economic, and medical reasons, compliance with restricted diets is often very poor, especially in the long term.

**Figure 1:**
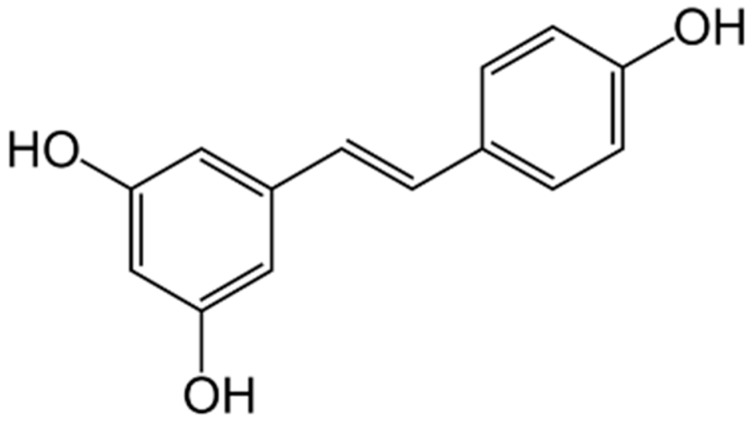
Chemical structure of *trans* resveratrol.

In this context, new molecules that could be used as dietary functional ingredients in the fight against obesity and its co-morbidities are constantly being sought. Among them, polyphenols represent a group of molecules of increasing interest. One of the most widely studied polyphenols is resveratrol (*trans*-3,4′,5-trihydroxystilbene) (Figure 1). This is a stilbene produced naturally in several plants in response to injury or fungal attack [9]. There are two different isoforms, *trans*-resveratrol and *cis*-resveratrol, but the *trans* form is the active one. Resveratrol has been proposed as an “energy restriction mimetic” because it can exert energy restriction-like effects [10,11].

The aim of this review is to analyze the effects of resveratrol on obesity under different feeding conditions, such as overfeeding, normal feeding, and energy restriction in animals and humans.

## 2. RSV Effects in Body Weight and Adipose Tissue Weight under Overfeeding Conditions in Preclinical Studies

The fundamental law of the energetics of obesity is that this condition can only develop when energy intake is greater than energy expenditure. In normal conditions, energy intake equals energy expenditure, which is the totality of different components: basal metabolic rate, physical activity, and thermogenesis [12].

In general, overfeeding rodents results in increased weight gain and body fat accumulation, although this response very much depends on the rodent strain [13,14,15]. Thus, high-fat diet feeding leads to increases in animal body weight and body fat percentage, as compared with those of the normal diet-fed counter partners, either in mice [16,17,18,19,20,21,22,23,24] or in rats [25,26,27] (Table 1). However, exceptions to this increase in body weight or fat accumulation when feeding a high-fat diet have also been reported [28]. In all these studies, fat percentages in the high-fat diets were highly variable, and ranged from 20% to 60% of diet energy.

The effects of resveratrol in preventing body-weight gain in animals fed high-fat diets are controversial. Thus, some authors found statistically significant body weight reductions in animals that were fed high-fat diets supplemented with resveratrol, either in mice [18,19,22,24,29,30,34] or rats [32]. Others saw only a tendency towards lower body weight [21,26], or did not report any change [20] after resveratrol supplementation. However, greater agreement is found when studying the effect of resveratrol on adipose depots in animals fed an obesogenic diet. Most studies reported a reduced size of adipose tissues, mainly in visceral fat depots (i.e., mesenteric, epididymal, perirenal and retroperitoneal), when animals were offered this polyphenol. These studies show that resveratrol is useful to prevent obesity, at least in rodent models.

The reductions reported in these studies were achieved in the presence of resveratrol at a broad range of doses (5 mg/kg/day to as much as 400 mg/kg/day). According to the published data, it is not as yet possible to determine the most effective dosage because a clear dose-response pattern is observed in some cases [31,32], while in other cases, a plateau is reached [31], or even greater responses are observed when using lower doses than when using higher ones [19]. Consequently, further research is needed to clarify this issue.

## 3. RSV Effects in Body Weight and Adipose Tissue Weight under Normal Feeding Conditions in Preclinical Studies

Although less widely studied, another situation in which resveratrol effects on body weight and adipose tissue weight has been analyzed, is its administration at the same time as a standard diet (Table 2). In the study reported by Lagouge et al. (2006), male C57BI/6J mice were fed a standard chow diet, with or without resveratrol (400 mg/kg body weight/day) for 15 weeks [29]. Under these conditions, resveratrol did not induce statistically significant changes in body weight. Unfortunately, no data concerning body fat weight are available in this paper. In the study reported by Mendes et al. (2016), female FVB/N mice were fed with a standard diet that was supplemented with resveratrol (300 mg/kg body weight/day) for 60 days [28]. No changes in body weight or adipose tissue weight induced by resveratrol administration were observed. Although these two studies were performed using different animal models (male C57BI/6J mice or female FVB/N mice), different experimental period lengths (8 to 15 weeks) and different resveratrol doses (300 to 400 mg/kg body weight/day), the results were similar. The lack of any resveratrol effect seems logical because healthy non-obese animals were used in the experiments, and resveratrol cannot prevent obesity development in a dietary situation that does not in fact promote excessive body fat accumulation.

Another scenario is where animals are first fed an obesogenic diet (diet-induced obesity) and once obese, are switched to a standard diet supplemented with resveratrol in order to know whether this phenolic compound is useful for obesity treatment. This experimental design is closer to that which is usually applied in obesity treatment in humans. In this regard, the only study in which these conditions have been analyzed was performed in our research group, as far as we know [35]. In this study, male Wistar rats were fed a high-fat high-sucrose diet for six weeks in order to induce obesity. Subsequently, animals were switched to a standard diet that was supplemented with resveratrol (30 mg/kg body weight/day), or not, for six additional weeks. When comparing body weight and adipose tissue weights of the animals in this group (standard diet + resveratrol) with the animals in the control group (standard diet alone), no differences were appreciated. The lack of effect observed under these experimental conditions suggests that an active phase of body fat accretion is needed by resveratrol to be effective.

Some studies under standard feeding conditions have been carried out in Zucker rats. When the studies were conducted in *fa*/*fa* Zucker rats, a model of genetic obesity, resveratrol induced significant reductions in body fat (−10%; [36]), or visceral adipose tissue weight (−14.9%; [37]). However, when the treatment was applied to lean Zucker rats (*Fa*/*Fa*), resveratrol treatment was ineffective. These results suggest once again that resveratrol needs a situation of excessive fat accumulation to be effective. This makes sense but, as we have described previously in this review, when diet-induced obese rats are treated with resveratrol within the framework of a standard normocaloric diet, its anti-obesity effects are not observed. Thus, a question arises: what is the difference between genetically obese Zucker rats that are fed a standard diet and diet-induced obese rats fed a standard diet? To explain this, it is important to point out that *fa*/*fa* Zucker rats show a genetic alteration, which, in turn, induces metabolic disturbances. These make animals increase body fat accumulation continuously throughout their life [38,39]. By contrast, rats that become obese due to overfeeding stop body fat accretion when they are switched to a normocaloric diet. As a result, as previously discussed in this review, resveratrol may need to be provided in a metabolic phase of active fat accumulation for its anti-obesity properties to be shown.

## 4. RSV Effects in Body Weight and Adipose Tissue Weight under Energy Restriction Conditions in Preclinical Studies

The effectiveness of energy restriction on body weight and adipose tissue weight reduction has been demonstrated in different animal models, as well as in humans [4]. Moreover, as explained before in this review, resveratrol has been proposed as an “energy restriction mimetic”, because it can exert energy restriction-like effects [10,11]. Bearing this in mind, the supplementation of energy restricted diets with resveratrol could be an effective tool for obesity management. In this regard, the hypothesis is that resveratrol could enhance the effects of energy restricted dietary pattern. This being the case, the reductions in body weight and body fat mass would be greater than the ones that are induced by energy restriction alone due to potential additive or synergistic effects.

Studies designed under these experimental conditions are also scarce in the literature (Table 3); two of the three studies reported have been carried out by our research group. Joseph et al. (2013) fed 27-month-old Fischer 344 × Brown Norway Hybrid rats a maintenance diet for six weeks while submitting the animals to 20% energy restriction and resveratrol supplementation (50 mg/kg body weight/day) or not [40]. At the end of the experimental period, energy restriction reduced body weight and body fat mass. Nevertheless, the addition of resveratrol to the restricted diet did not induce greater changes in the aforementioned parameters. In the first study carried out by our group, we fed six-week-old male Wistar rats a high-fat high-sucrose diet (HFHS) for six weeks in order to induce obesity. Then, animals were fed a standard diet for two additional weeks and were submitted to 25% energy restriction with resveratrol supplementation (30 mg/kg body weight/day) or not. At the end of the experiment, differences were observed between the control group and both restricted groups regarding body weight and different adipose depot weights. However, as in the case of Joseph et al., no different weight and fat reductions were appreciated when energy restriction was used alone or in combination with resveratrol [41]. In this study, we chose 25% energy restriction because it is commonly used in interventions that are conducted in humans, and a dose of 30 mg resveratrol/kg body weight/day because this dose was effective in previous studies from our group [31]. The absence of effects observed on this study suggested the possibility that strong effects of energy restriction could mask those of resveratrol. In order to avoid this possible bias, in a second study we used the same experimental design, but a lower energy restriction percentage (15%). The results obtained in this study were similar to those of our previous study and to those reported by Joseph et al. [40]. Thus, energy restriction reduced body and adipose tissue weights when comparing with a control group, but resveratrol addition did not induce any effect on these changes [35].

These three studies confirmed that energy restriction is effective in reducing body weight and adipose tissue weight under different experimental conditions: energy restriction degree (15%, 20% or 25%), experimental period length (two or six weeks), rat strain (Fischer 344 × Brown Norway Hybrid or Wistar rats), and animal age (six weeks or 27 months). However, the administration of resveratrol in the range of 30 to 50 mg/kg body weight/day, together with restricted diets (15% to 25% energy restriction) did not induce additional reductions in body weight or adipose tissue weights with regard to those induced by energy restriction. The reason that justifies this lack of effect is not the doses of resveratrol used, because resveratrol in the range of 30 to 50 mg/kg body weight/day has been demonstrated to be effective under overfeeding conditions [42,43].

## 5. RSV Effects in Body Weight and Body Fat Weight in Clinical Studies

Studies addressed in humans devoted to analyzing the potential anti-obesity effect of resveratrol are scarce to date [44]. Moreover, some of them have been directed in healthy non-obese subjects [45,46,47]. In addition, the vast majority of them do not describe the dietary pattern that is followed by the subjects that are involved in the study [45,46,48,49,50,51,52,53,54]. Among the twelve clinical trials analyzed in this review, only three described diet composition.

Van der Made et al. (2015) performed a placebo-controlled crossover study in overweight or obese men and women [55]. Subjects received 150 mg/day of resveratrol for four weeks, and the diet was similar in terms of energy (2273 kcal/day and 2373 kcal/day), carbohydrates (46% and 45%), protein (16% and 15%), total fat (36% and 38%), alcohol (2% for both groups), fiber (25 g/day for both groups), and cholesterol (201 mg/day and 202 mg/day) in placebo and resveratrol groups, respectively. As far as fat composition is concerned, monounsaturated fatty acids represented 12% and 13% of energy intake in control and resveratrol-treated groups, respectively. By contrast, no differences were observed in saturated fatty acids (12% of energy intake), or polyunsaturated fatty acids (9% of energy intake). Under these experimental conditions, body weight remained unchanged after resveratrol treatment.

In another study, 192 patients were randomly distributed into three groups: (a) placebo group; (b) a group treated with 40 mg resveratrol/day; and (c) a group treated with 500 mg resveratrol/day for six months. They maintained their habitual lifestyle and the diet was (<2400 kcal/day; 45–60% carbohydrate, <10% sugars; <35% fat, <10% saturated fat; 10–20% protein; 20 g/1000 kcal fibre; <6 g/day salt). All of the patients kept to their current hypoglycemic treatment during the trial, but avoided using nutritional supplements or consuming significant amounts of resveratrol-rich foods and beverages. Once again, no significant differences were found in body weight, body mass index, or waist circumference [56].

More recently, a randomized double-blind, placebo-controlled clinical trial has been reported. The patients suffered type 2 diabetes mellitus, and a body mass index of 18.5–30 kg/m^2^. The experimental groups received either 480 mg resveratrol/day or placebo (starch) for four weeks. No differences were observed for dietary intake, including energy intake (≈1800 kcal/day and macronutrients (50–55% carbohydrate; <35% fat; 10–20% protein) at baseline or after the treatment. Resveratrol treatment did not modify body weight, BMI, waist circumference, or hip circumference [47].

As it can be observed, clinical trials are performed with balanced macronutrient composition diets. Due to the fact that these studies did not provide the energy expenditure of the patients, we cannot define whether diets were hypercaloric, normocaloric, or hypocaloric. Nevertheless, hypercaloric diets can be discarded because it is not ethical to propose them to obese subjects. Consequently, diets should be either normocaloric or hypocaloric. As previously explained in this review, under these experimental conditions resveratrol was also ineffective in reducing body fat in preclinical studies. Thus, results in humans give support to our hypothesis concerning the need for an active phase of body fat accretion if resveratrol is to have an effect on body fat.

## 6. Metabolic Background

**Figure 2:**
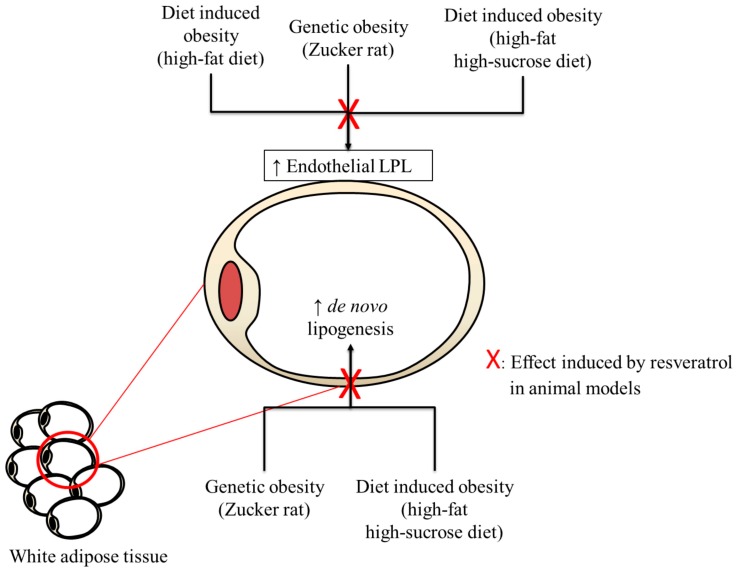
Alterations induced by dietary and genetic obesity in white adipose tissue triacylglycerol metabolism and resveratrol actions.

In order to give an explanation for the reported effects of resveratrol under different feeding conditions, the following paragraphs will describe the changes in adipose tissue triacylglycerol metabolism that are present in genetically obese animals or induced by obesogenic diets, as well as the modifications induced by resveratrol in these metabolic pathways (Figure 2).

Obese Zucker rat show several alterations in white adipose tissue triacylglycerol metabolism. These included increased activity of lipoprotein lipase (LPL), the enzyme that hydrolyses triacylglycerols circulating as chylomicrons, and very low density lipoproteins (VLDL) into free fatty acids, and increased lipogenic enzyme activity, involved in de novo lipogenesis, the metabolic process that allows adipose tissue to synthesize fatty acids from acetyl-CoA. These metabolic alterations, among others, lead to increased body fat accumulation from a very early age in these particular rats [38,57]. With regard to animals that were fed a high-fat diet, it has been reported that this dietary pattern leads to increased LPL activity in the long term [58]. In the case of high-fat high-sucrose diets, both (increased LPL activity and de novo lipogenesis) are observed [59]. As a result, in both cases, increased availability of free fatty acids in adipose tissue leads to their enhanced storage as triglycerides. When resveratrol is administered to the animals, it is able to inhibit LPL [33,37,60] and lipogenic enzymes [18,19,22,33,60], thus counteracting, at least in part, the metabolic alterations that lead to increased fat accretion in both genetic and dietary-induced obesity models. Consequently, resveratrol acts in preventing obesity development.

Szkudelska et al. (2009) reported an enhanced lipolytic response to epinephrine in adipocytes that are exposed to resveratrol [61]. However, this phenolic compound failed to raise basal glycerol release. Moreover, ATGL gene expression was increased in both 3T3-L1 and human SGBS (Simpson Golabi Behmel Syndrome) adipocytes that were incubated with resveratrol [62]. Furthermore, in vivo studies carried out in our laboratory showed increased gene expression of hormone sensitive lipase (HSL) in rats fed a high-fat diet and treated with resveratrol [60], and also in genetically obese rats that were fed a standard diet [37]. In view of these results, a reduction in body fat in animals treated with resveratrol under normal feeding should be expected, but in fact, this was not the case.

When animals are submitted to energy restriction, greatly increased lipolysis is induced [63]. Bearing this is mind, and considering the results reported by Szkudelska et al. (2009), increased lipolysis, and thus, enhanced body fat loss, should be expected in animals receiving resveratrol during energy restriction [61]. But, once again, this effect was not observed. In order to understand this lack of effect, it is important to point out that the results that were reported by Szkudelska et al. (2009) and Lasa et al. (2012) were obtained in cultured cells, and perhaps in vitro results may not be reproduced in vivo [61,62]. Moreover, in the study reported by Lasa et al. (2012) the amounts of resveratrol used (100–200 μM) were far higher than those found in blood and tissues after resveratrol administration [62]. With regard to our studies where we found increased HSL gene expression, it is important to remember that the main regulatory mechanism of lipolysis is lipase phosphorylation. Thus, the lipolytic effect of resveratrol requires further studies. Taken together, these results can help to understand the absence of any resveratrol body fat-lowering effect under normal feeding or energy restriction feeding conditions.

## 7. Concluding Remarks

After analyzing the reported results that were obtained under overfeeding, normal feeding, and energy restriction feeding conditions, it can be stated that resveratrol is useful to reduce body fat accumulation, and thus to prevent obesity. However, for ethical reasons, these results have been obtained in animals. By contrast, there is no evidence of its usefulness in reducing previously accumulated body fat. Consequently, as of yet, there is not scientific support to propose resveratrol as a new anti-obesity treatment tool. Nevertheless, it is important to point out that resveratrol may induce favorable effects on several obesity co-morbidities, such as insulin resistance and fatty liver, without changes in adiposity.

## Figures and Tables

**Table 1:** Preclinical studies performed administering resveratrol under overfeeding conditions.

Author	Animal Model	Diet	Increase in Dietary Fat (vs. Control)	Lower Body Weight	Lower Fat Weight
Lagouge et al., 2006 [29]	C57BL/6J mice	High-fat diet (40% fat)	31.6%	Yes	Visceral Yes Subcutaneous Yes
Baur et al., 2006 [16]	C57BL/6NIA mice	High-fat diet (60% fat)	40%	No	No
Kim et al., 2011 [18]	C57BL/6J mice	High-fat diet (40% fat)	28%	Yes	Visceral Yes Subcutaneous N/A
Kang et al., 2012 [20]	C57BL/6N mice	High-fat diet (58% fat)	45%	No	N/A
Cho et al., 2012 [19]	C57BL/6J mice	High-fat diet (40% fat)	29%	Yes	Visceral Yes Subcutaneous N/A
Wang et al., 2013 [21]	C57BL/6J mice	High-fat diet (44% fat)	31%	No	N/A
Jeon et al., 2014 [30]	Homozygous apoE-deficient mice	Atherogenic diet (20% fat)	No control diet fed group	Yes	Visceral Yes Subcutaneous N/A
Qiao et al., 2014 [22]	Kunming mice	High-fat diet (50% fat)	40%	Yes	Yes Location data N/A
Carpéné et al., 2014 [23]	C57BL/6J wild-typemice	Very-high-fat diet (60% fat)	50%	No	No
Montero et al., 2014 [24]	C57BL mice	High-fat diet (60% fat)	50%	Yes	N/A
Shang et al., 2008 [26]	Wistar rats	High-fat diet (59% fat)	STD diet composition N/A	Yes	Visceral Yes Subcutaneous N/A
Macarulla et al., 2009 [31]	Sprague-Dawley rats	Obesogenic diet (45% fat)	No control diet fed group	No	Visceral Yes Subcutaneous Yes
Arias et al., 2011 [32]	Wistar rats	Obesogenic diet (45% fat)	No control diet fed group	Yes	Visceral Yes Subcutaneous No
Poulsen et al., 2012 [27]	Wistar rats	High-fat diet (60% fat)	50%	No	Visceral No Subcutaneous N/A
Arias et al., 2014 [33]	Wistar rats	Obesogenic diet (45% fat)	No control diet fed group	No	Visceral No Subcutaneous No

N/A: not available.

**Table 2:** Preclinical studies performed administering resveratrol under standard conditions.

Author	Animal Model	Diet	Lower Body Weight	Lower Fat Weight
Lagouge et al., 2006 [29]	C57BL/6J mice	Chow diet (8% lipid, 19% protein, 73% carbohydrate)	No	N/A
Mendes et al., 2016 [28]	FVB/N mice	Standard diet (12% lipid, 23% protein, 65% carbohydrate)	No	No
Milton-Laskibar et al., 2017 [35]	Wistar rats	Standard diet (16% lipid, 20% protein, 64% carbohydrates)	No	No

N/A: not available.

**Table 3:** Preclinical studies performed administering resveratrol under energy restriction conditions.

Author	Animal Model	Diet	Energy Restriction Degree (vs. Control)	Lower Body Weight	Lower Fat Weight
Joseph et al., 2013 [40]	Fischer 344 × Brown Norway Hybrid rats	Standard maintenance diet (10% lipid, 14% protein, 76% carbohydrate)	20%	No	No
Alberdi et al., 2014 [41]	Wistar rats	Standard diet (16% lipid, 20% protein, 64% carbohydrate)	25%	No	No
Milton-Laskibar et al., 2017 [35]	Wistar rats	Standard diet (16% lipid, 20% protein, 64% carbohydrate)	15%	No	No
